# Evaluation of DNA/Chromosome Integrity and Cell Death in Human Metabolically Noncompetent and Competent Cells Exposed to *N*′‐(3,5‐Difluorobenzylidene)Pyridine‐4‐Carbohydrazide

**DOI:** 10.1002/jat.70171

**Published:** 2026-03-27

**Authors:** Larissa Ribeiro Canuto Santos, Jéssica Natalia Barbosa de Almeida, Camila Cavalleiro Frias, Wanda Pereira Almeida, Edson Luis Maistro

**Affiliations:** ^1^ Faculty of Philosophy and Sciences, Speech and Hearing Therapy Department São Paulo State University – UNESP Marília São Paulo Brazil; ^2^ Faculdade de Medicina de Marilia FAMEMA Marília São Paulo Brazil; ^3^ Instituto de Química Universidade Estadual de Campinas Campinas São Paulo Brazil; ^4^ Laboratório de Pesquisa em Fármacos e Medicamentos, Faculdade de Ciências Farmacêuticas Universidade Estadual de Campinas Campinas São Paulo Brazil

**Keywords:** acylhydrazones, Alzheimer treatment, comet assay, HepG2/C3A cells, micronucleus test, risk assessment

## Abstract

The N‐acylhydrazone scaffold is recognized as a privileged structure for the design of bioactive substances with increasing applications in medicinal chemistry research. Ensuring the safety of newly developed molecules is a critical step for both human health and environmental protection. Accordingly, this study aimed to evaluate the cytotoxic and genotoxic properties of *N*′‐(3,5‐difluorobenzylidene)pyridine‐4‐carbohydrazide in two cellular models: nonmetabolizing leukocytes and metabolically active hepatic cells (HepG2/C3A). The resazurin‐based cytotoxicity analysis, performed with concentrations between 1 and 600 μg/mL, indicated that only the uppermost concentration caused a marked decrease in viability of both cell populations after 48 h of incubation. Regarding genotoxicity at 50, 100, and 200 μg/mL concentrations, no DNA damage was detected in the comet assay, but in the micronucleus test, a significant increase in chromosome alterations in leukocytes at 200 μg/mL concentration was detected, with a decrease in cell proliferation in both cell types. The data indicate that, at the concentrations where the biological effects of acylhydrazone were previously observed, the substance appeared to be safe, but at higher concentrations and/or during chronic exposure, caution and further studies are needed.

## Abstract 2

This study investigated the cytotoxic and genotoxic effects of *N*′‐(3,5‐difluorobenzylidene)pyridine‐4‐carbohydrazide in two human cell models: leukocytes and HepG2/C3A. Cytotoxicity assays performed with concentrations ranging from 1 to 600 μg/mL revealed a reduction in cell viability only at the highest concentration, after 48‐h exposition. Regarding genotoxicity, exposure to 50, 100, and 200 μg/mL did not induce DNA damage in the comet assay. However, in the micronucleus test, a significant increase in chromosomal alterations was observed in leukocytes at 200 μg/mL.

## 1 Introduction

The acylhydrazones are a class of organic compounds that, because of their structure, have significant malleability both chemically and pharmacutically. The structural variability of these molecules offers the possibility to synthesize compounds with various therapeutic properties, as Alzheimer treatment, cancer, anticonvulsant, anti‐inflammatory, antihypertensive, antioxidant, cytotoxic, antiparasitic, antibacterial, antifungal, and antiviral (Brum et al. 2020; Mali et al. 2021).

When considering the synthesis and development of compounds with biological activity and as electrophiles for the synthesis of nitrogenous substances, the N‐acylhydrazone group is a versatile structure with potential applications in various branches of chemistry and biology (Sugiura and Kobayashi 2005; Thota et al. 2018).

In this context, Frias et al. (2024) synthesized and characterized the compound *N*′‐(3,5‐difluorobenzylidene)pyridine‐4‐carbohydrazide. The authors observed that this synthetic acylhydrazone exhibited an IC_50_ value for acetylcholinesterase (AChE) inhibition of 2.73 μM (0.71 μg/mL). This biological effect makes this molecule an adjuvant candidate to treat conditions like Alzheimer's disease and myasthenia gravis by increasing acetylcholine levels, thereby enhancing cholinergic neurotransmission (AlNasser et al. 2025; Saud et al. 2024).

Acylhydrazones are a versatile class of organic compounds widely studied for their biological activity, but their toxicity profile is complex and depends strongly on structural variations. Generally, acylhydrazones can interact with biological systems due to their ability to form stable hydrogen bonds and coordinate with metal ions, which contributes to both therapeutic potential and toxicological risks. Some derivatives have shown cytotoxicity by interfering with mitochondrial function and inducing oxidative stress. Their toxicity is often linked to the generation of reactive intermediates that can damage cellular components, particularly DNA and proteins. Importantly, while certain acylhydrazones are being explored as drug candidates, careful evaluation of dose‐dependent effects and structural modifications is essential to minimize adverse outcomes and ensure safety in pharmacological applications (Socea et al. 2022).

Carcinogenesis refers to the sequence of events leading to cancer, beginning with the malignant transformation of cells triggered by chemical exposure. It involves disruptions in genomic stability caused by gene mutations and chromosomal damage, ultimately resulting in tumor formation. Understanding the link between DNA damage and cancer onset is essential in the preclinical safety evaluation of pharmaceuticals and chemical agents. Such assessments focus on determining the genotoxic potential of xenobiotics and their capacity to induce DNA alterations and carcinogenic effects (Secerli et al. 2024; Ellinger‐Ziegelbauer et al. 2009).

Considering in vitro assays for the determination of cytogenotoxicity, human peripheral blood lymphocytes are relevant peripheral cell types that reflect systemic exposure and genomic stability (Antoniou et al. 2023). The use of lymphocytes as a model provides an appropriate correlation between in vitro findings and real‐world human exposure, thereby supporting the extrapolation to potential occupational and dietary risks (Lebailly et al. 2015). Additionally, the human hepatoblastoma cell line HepG2 has been used for more than 15 years in mutagenicity studies. Unlike many other cell lines employed in genetic toxicology, HepG2 cells have preserved the activity of several phase I and phase II enzymes, which are essential for the activation and detoxification of promutagens and procarcinogens (Knasmüller et al. 2004; Hewitt and Hewitt 2004).

As *N*′‐(3,5‐difluorobenzylidene)pyridine‐4‐carbohydrazide represents a newly synthesized acyl hydrazone, this research was designed to assess its potential cellular and genetic toxicity. The compound was tested in two distinct models: human leukocytes, which lack hepatic metabolic activity, and HepG2/C3A hepatoma cells, which possess metabolic competence.

## 2 Material and Methods

### 2.1 The Test Compound

The acylhydrazone under investigation, designated as *N*′‐(3,5‐difluorobenzylidene)pyridine‐4‐carbohydrazide, Figure 1, is available in our laboratory and was synthesized and characterized as previously described (Frias et al. 2024). Briefly, the synthetic route involved the reaction of 3,5‐difluorobenzaldehyde with 4‐pyridinecarboxylic acid hydrazide, affording the acylhydrazone as a pale yellow solid. The compound was purified by recrystallization from EtOH–hexane, yielding 71%. For the experiments, 10 mg of acylhydrazone was dissolved in 100 μL of DMSO and subsequently diluted with 9.9 mL of sterile distilled water, yielding a working solution with a concentration of 1000 μg/mL.

**FIGURE 1:**
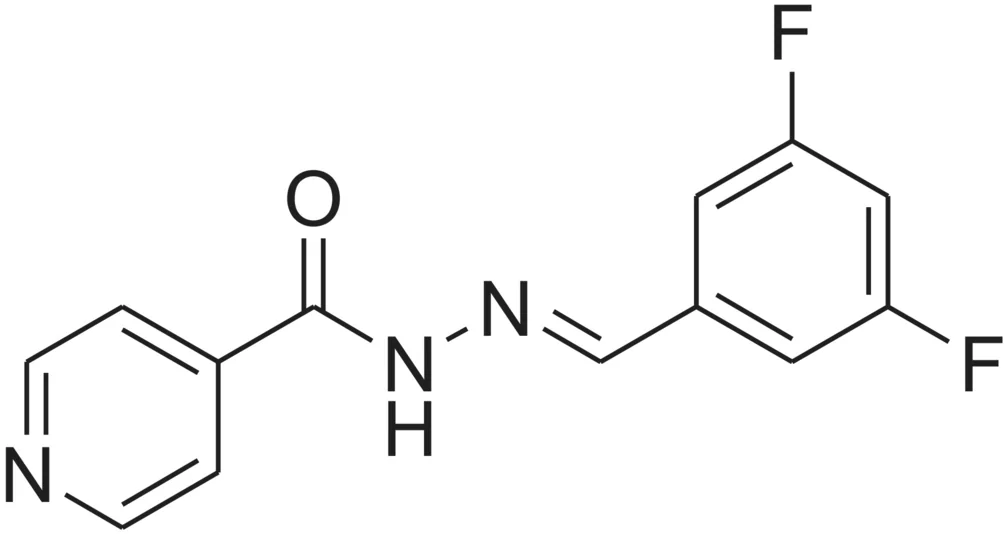
Chemical structure of *N*′‐(3,5‐difluorobenzylidene)pyridine‐4‐carbohydrazide.

### 2.2 Ethical Issues

Approval for this research was granted by the Research Ethics Committee responsible for studies involving human participants at UNESP, Marília Campus, on October 7, 2024, under CAAE protocol number 81227824.5.0000.5406. Blood samples were drawn from male and female adult volunteers who agreed to participate by signing the Free and Informed Consent Form, in accordance with the guidelines of Resolution 466/2012 issued by the Brazilian National Health Council.

### 2.3 Cell Culture Conditions

Two distinct cellular systems were applied in this work, in compliance with OECD chemical testing guidelines (OECD 489 2016a; OECD 487 2016b). Human PBMCs, lacking hepatic metabolic capacity, were isolated from two healthy young adults (18–35 years), representing both sexes, nonsmokers, and drug‐free. Cultures were grown in RPMI 1640 medium containing 10% fetal bovine serum (Gibco) and 1% antibiotic/antimycotic mixture (Gibco), thereby constituting complete RPMI. For the lymphocyte micronucleus test, 5 μg/mL phytohemagglutinin (Sigma‐Aldrich) was added to trigger proliferation and mitotic entry. Incubation was performed at 37°C under humidified conditions with 5% CO_2_ and 95% relative humidity.

HepG2/C3A hepatocarcinoma cells, noted for their active hepatic metabolic enzyme profile, were cultured as monolayers in 25‐cm^2^ flasks containing high‐glucose DMEM (Gibco) supplemented with 10% fetal bovine serum (FBS, Gibco) and 1% antibiotic/antimycotic solution (Gibco), designated as complete DMEM. Cultures were incubated at 37°C under humidified conditions with 5% CO_2_. Proliferation was monitored daily using an inverted optical microscope, and medium replacement was performed when cells neared confluence or nutrient renewal was required. Trypsin (Sigma‐Aldrich) was applied for detachment. At ≥ 80% confluence, cells were transferred to 75‐cm^2^ flasks and maintained for a minimum of 5 days to reach the density needed for experimental procedures. The HepG2/C3A cell line was obtained from the Rio de Janeiro Cell Bank, Federal University of Rio de Janeiro (UFRJ‐Brazil). In all experiments, cells were seeded into plates and treated with the test compound after a 24‐h stabilization period.

### 2.4 Cytotoxicity Assay

Considering that this acylhydrazone exhibits an IC50 value of 2.73 μM (0.71 μg/mL) for AChE (AChE) inhibition, concentrations of 1, 10, 100, 300, and 600 μg/mL were selected for cytotoxicity assessment in PBMC and HepG2/C3A cells using the resazurin assay. The procedure was carried out according to the manufacturer's instructions. Cells were seeded in 96‐well plates at a density of 2.5 × 10^4^ cells per well, with four replicates per concentration, in 100 μL of medium supplemented with FBS. After a 24‐h stabilization period, cultures were exposed to the test compound for 24 and 48 h, reaching a final volume of 200 μL. Positive and negative controls were included to ensure experimental validity. Triton X‐100 at 2% in serum‐free medium served as the positive control, untreated cells as the negative control, and serum‐free medium alone as the blank. Following treatment, the medium was removed and replaced with 100 μL of resazurin solution (60 μM in DMEM without FBS), followed by incubation at 37°C for 3 h. Fluorescence was measured using a spectrophotometric microplate reader equipped with a 520‐nm excitation filter (Epoch‐Biotek). Cell viability was expressed as a percentage, calculated using the formula: viability (%) = [(mean fluorescence of treated cells − blank absorbance) / (mean fluorescence of control cells—blank absorbance)] × 100.

### 2.5 Comet Assay

The genotoxic potential evaluation of the studied compound on PBMC and HepG2/C3A cells was assessed by the comet assay, following the protocol described by Tice et al. (2000). Initially, microscope slides were prepared by applying a thin and homogeneous coating of normal melting point (NMP) agarose. In a 12‐well plate, 2 × 10^5^ cells were added and maintained for 24 h for cell attachment to the culture flask. Considering that up to a concentration of 200 μg/mL no cytotoxic effect of the test compound was observed in the resazurin test, and that its IC_50_ for inhibition of AChE is 0.71 μg/mL (Frias et al. 2024), concentrations of 50, 100, and 200 μg/mL were chosen to perform the genotoxicity/mutagenicity tests of the compound. After 4 h of treatment, cells were harvested and 20 μL of medium with the cells were mixed with 120 μL of low melting point (LMP) agarose, and deposited on the microscope slide previously precoated with NMP agarose, and immediately covered with a coverslip. Afterward, the slides were taken to the refrigerator for 30 min. After removing the coverslips, the slides were placed in a lysis solution (2.5 M NaCl, 100 mM EDTA, 10 mM Tris, 1% Triton x‐100, and 10% DMSO [pH 10]), where they remained overnight. Following cell lysis, slides were rinsed with PBS, placed in an electrophoresis chamber, and immersed in alkaline buffer (pH > 13; 300 mM NaOH and 1 mM EDTA) for 20 min to promote DNA denaturation. Electrophoresis was subsequently carried out in an ice bath at 4°C for 20 min under conditions of 25 V and 300 mA (0.722 V/cm). After completion, slides were withdrawn from the chamber and incubated in neutralization buffer (0.4 M Tris–HCl, pH 7.5) for 15 min. They were then air‐dried at room temperature, fixed with absolute ethanol for 10 min, and stored in slide boxes under refrigeration until further analysis. For the analysis, the gels were stained with 100 μL of ethidium bromide solution and then covered with a coverslip. A fluorescence microscope (Olympus BX‐50), with a 515–560 nm excitation filter and a 590‐nm barrier filter, was used for the visual analysis of the nucleoids, using a 40× objective. The experiments were performed in triplicate for PBMC and HepG2/C3A cells.

One hundred randomly selected nonoverlapping nucleoids (50 cells per coded slide) were analyzed per culture well. According to the size of the tail, the nucleoids were classified into the following four classes: class 0—no tail (damage 0); class 1—tail shorter than the diameter of the head (damage 1); class 2—tail length 1–2 times the head diameter (damage 2); and class 3—tail length more than twice the diameter of the head (damage 3). Comets without defined heads, with almost all DNA in the tail or with a large tail were classified as damage 4, and scored separately, because they likely represent cells that have undergone apoptosis. The scores of the total of 100 nucleotides, which can vary from 0 (no damage) to 300 (severe damage), were obtained from the sum of the multiplication of the number of nucleotides in each class by the damage class (Hartmann and Speit 1997; OECD 489 2016a).

### 2.6 Cytokinesis‐Block Micronucleus Assay

Cytokinesis‐block micronucleus (CBMN) assay was executed in accordance with the methodology proposed by Fenech (2000) and the OECD 487 guideline (2016). Experiments with donor‐derived leukocytes were performed in duplicate, resulting in four replicates overall, while HepG2/C3A cultures were assessed in triplicate. The cultured cells were maintained in 25‐cm^2^ culture flasks (TPP). For leukocyte assays, 150 μM methyl methanesulfonate (MMS) was employed as the positive control, whereas 2 μM benzo(a)pyrene was used for HepG2/C3A cells. Whole blood samples (0.5 mL) were diluted in 4.5 mL of culture medium supplemented with 10% fetal bovine serum (FBS) and 1% antibiotic/antimycotic. Phytohemagglutinin (PHA) was added at 10 μL/mL to stimulate lymphocyte proliferation, and cultures were maintained for 72 h at 37°C in a humidified incubator with 5% CO_2_. At 44 h, cells were treated with the test acylhydrazone at concentrations of 50, 100, and 200 μg/mL. Four hours later (*T* = 48 h), cytochalasin‐B (6 μg/mL) was introduced into each culture. After 72 h, cells were collected by centrifugation (5 min at 850 g) and resuspended in 0.075 M KCl hypotonic solution for 5 min. To terminate hypotonization, four drops of a methanol/acetic acid solution (5:4) were added, and the samples were centrifuged. The pellet was resuspended in ice‐cold methanol/acetic acid (5:4), with three drops of 1% formaldehyde incorporated to preserve cytoplasmic structures. After centrifugation, fixation was repeated using methanol/acetic acid at a 3:1 ratio. Small aliquots of the suspension were then applied to slides, air‐dried at ambient temperature, and stained with 10% Giemsa in phosphate buffer (pH 6.8) for 10 min prior to microscopic examination.

HepG2/C3A cells were cultured for a complete cell cycle (24 h) and then treated with the studied acylhydrazone for a period of 24 h. Then, the cells were washed, harvested, and again incubated with cytochalasin‐B for 28 h. Afterward, the same procedure described above for leukocytes was followed.

The analyses of the slides were performed with an optical light microscope (Zeiss, Primo Star), with 40× or 100× amplification. A total of 1000 binucleated cells per flask were analyzed, and the frequencies of micronucleated cells, nucleoplasmic bridges, and nuclear buds were recorded. Cytotoxicity was quantified using the cytokinesis‐block proliferation index (CBPI), defined as CBPI = (M1 + 2M2 + 3M3) / *N*. In this formula, M1–M2 indicate the counts of cells with one or two nuclei, M3 represents those with more than two nuclei, and N (set at 500) is the number of evaluated cells (OECD 487 2016b).

### 2.7 Statistical Analysis

Following confirmation of data normality, statistical evaluation was conducted using analysis of variance (ANOVA), with Dunnett's and/or Tukey's post hoc comparisons applied as appropriate. All analyses were performed with GraphPad Prism Version 5, and differences were regarded as statistically significant when *p* < 0.05.

## 3 Results

Figures 2 and 3 illustrate the results of cell viability using resazurin assay for PBMC and HepG2/C3A cells, respectively, treated with the tested acylhydrazone for 24 and 48 h at concentrations ranging from 1 to 600 μg/mL. As can be seen, the compound did not markedly affect cell viability at any of the tested concentrations after 24‐h exposure. However, after 48‐h exposure, at the concentration of 600 μg/mL a slight but significant decrease in cell viability was observed. As expected, Triton X‐100 (2%) used as positive control significantly reduced cell viability of both cells.

**FIGURE 2:**
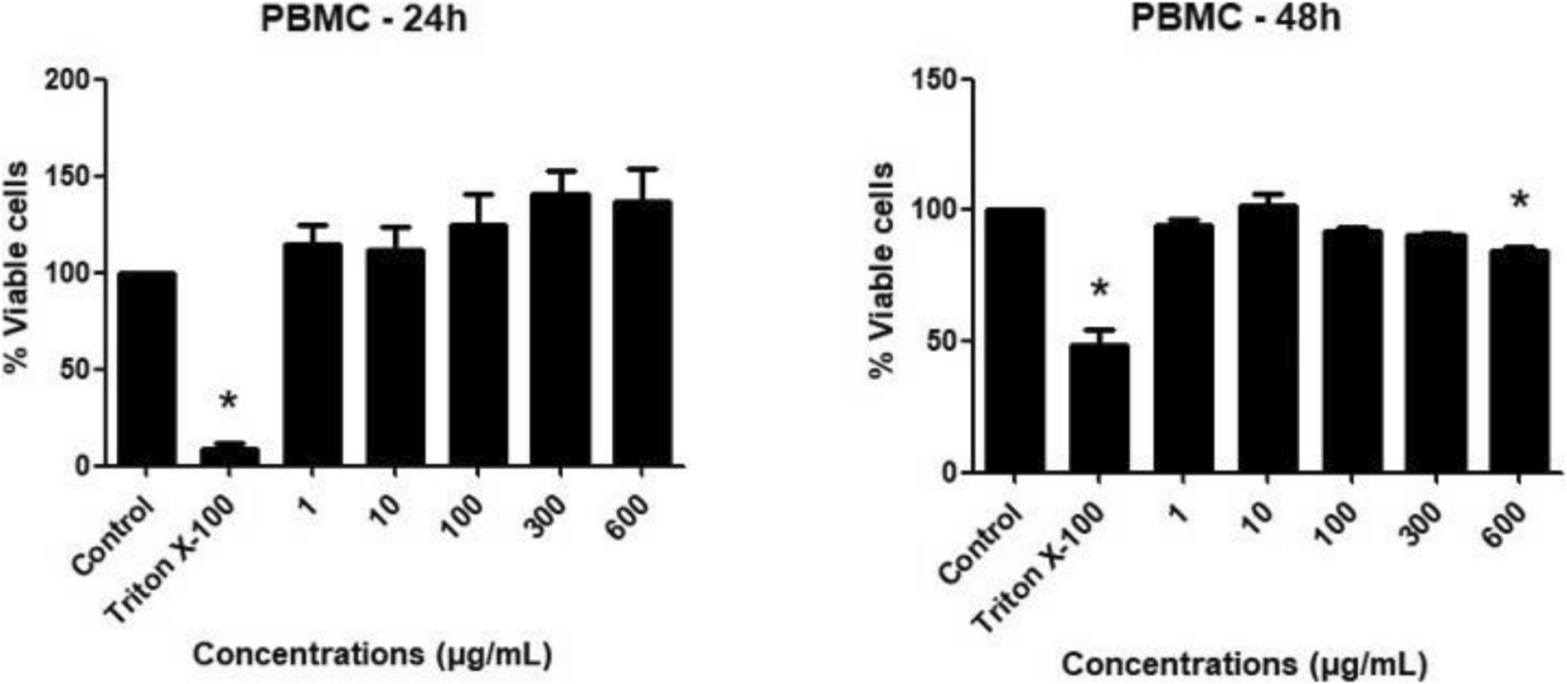
Percentage of viable PBMC cells after 24 and 48 h of exposure to different concentrations of *N*′‐(3,5‐difluorobenzylidene)pyridine‐4‐carbohydrazide, evaluated by the viability test with resazurin. Significantly different from the negative control: *p < 0.05.

**FIGURE 3:**
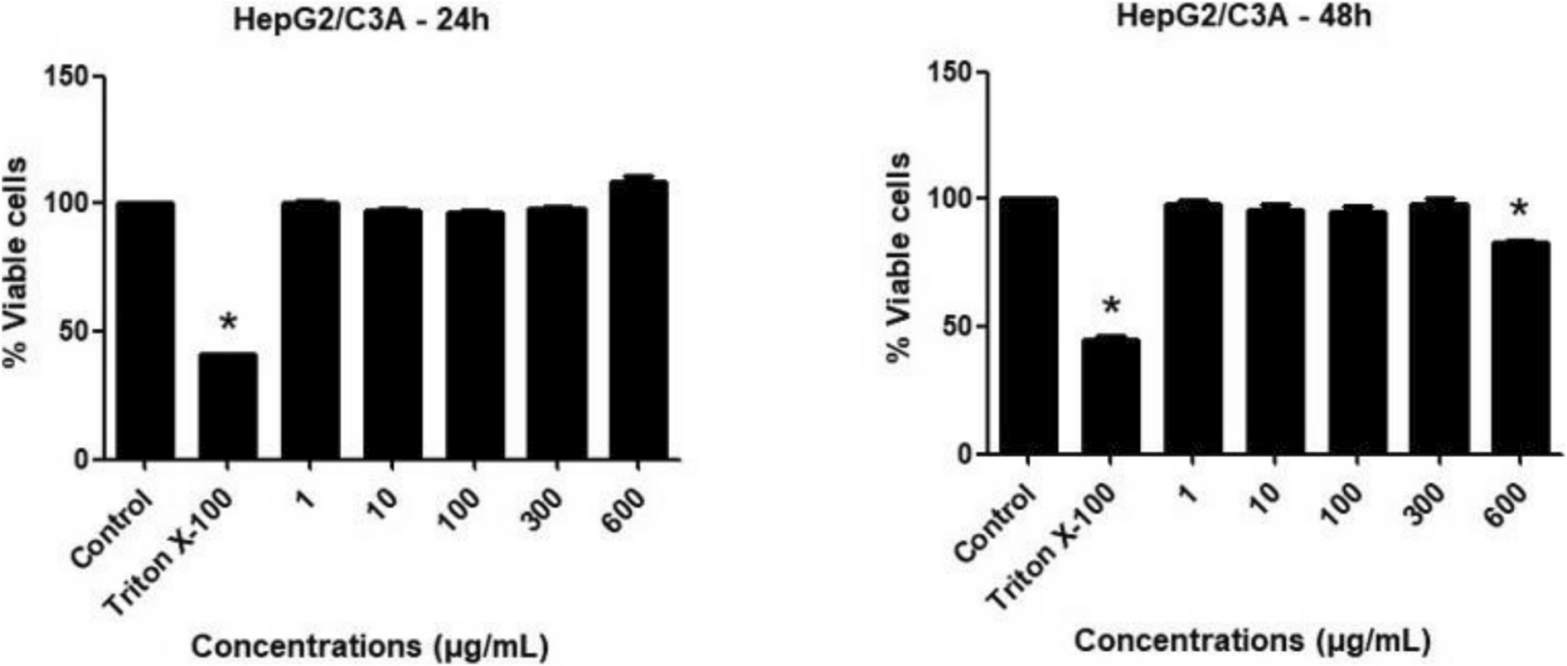
Percentage of viable HepG2/C3A cells after 24 and 48 h of exposure to different concentrations of *N*′‐(3,5‐difluorobenzylidene)pyridine‐4‐carbohydrazide, evaluated by the viability test with resazurin. Significantly different from the negative control: *p < 0.05.

Tables 1 and 2 display the impact of single treatment with *N*′‐(3,5‐difluorobenzylidene)pyridine‐4‐carbohydrazide and methyl methane‐sulfonate (MMS) on the DNA of PBMC and HepG2/C3A human cells. None of the tested concentrations caused any significant alteration in the total number of damaged cells and damage scores for both cell types in comparison with the control group. In the few nucleoids where DNA damage was detected, the majority of the damage was minor and categorized as class 1. MMS induced a significant increase in DNA damage in both cell types, confirming the sensitivity of the comet assay for the detection of DNA damage.

**TABLE 1:** DNA damage assessed by comet assay of *N*′‐(3,5‐difluorobenzylidene)pyridine‐4‐carbohydrazide in PBMC cells, after 4 h of treatment.

Comet assay						
Treatments	Total^a^	0	1	2	3	Scores
**Control**	3.50 ± 1.23	96.50 ± 1.25	3.00 ± 1.55	0.50 ± 0.57	0.00 ± 0.00	4.00 ± 1.63
**Test hydrazide (50 μg/mL)**	3.66 ± 0.57	97.25 ± 1.89	2.75 ± 1.89	0.00 ± 0.00	0.00 ± 0.00	2.75 ± 1.89
**Test hydrazide (100 μg/mL)**	2.75 ± 1.70	97.25 ± 1.70	2.75 ± 1.70	0.00 ± 0.00	0.00 ± 0.00	2.75 ± 1.70
**Test hydrazide (200 μg/mL)**	4.50 ± 2.08	95.50 ± 2.08	4.50 ± 2.08	0.00 ± 0.00	0.00 ± 0.00	4.50 ± 2.08
**MMS (45 μM)**	57.50 ± 4.73*	42.50 ± 4.73*	56.50 ± 4.20*	1.00 ± 0.81	0.00 ± 0.00	58.50 ± 5.44*

*Note:* DNA damage was expressed as “arbitrary units.” Control = DMSO 1%. One‐way analysis of variance and Dunnett's test.

^a^ Total number of damaged cells (class 1 + 2 + 3). Mean ± standard deviation of four replicates.

*Significantly different from negative control: *p < 0.05.

**TABLE 2:** DNA damage assessed by comet assay of *N*′‐(3,5‐difluorobenzylidene)pyridine‐4‐carbohydrazide in HepG2/C3A cells, after 4 h of treatment.

Comet assay						
Treatments	Total^a^	0	1	2	3	Scores
**Control**	2.33 ± 2.08	97.67 ± 2.08	2.33 ± 2.08	0.00 ± 0.00	0.00 ± 0.00	2.33 ± 2.08
**Test hydrazide (50 μg/mL)**	9.00 ± 1.00	91.00 ± 1.00	7.00 ± 1.00	2.00 ± 1.00	0.00 ± 0.00	11.00 ± 1.73
**Test hydrazide (100 μg/mL)**	7.00 ± 2.64	91.67 ± 4.93	6.00 ± 2.00	0.66 ± 0.57	0.33 ± 0.57	8.33 ± 4.04
**Test hydrazide (200 μg/mL)**	5.33 ± 1.15	94.67 ± 1.15	5.00 ± 1.73	0.33 ± 0.57	0.00 ± 0.00	5.66 ± 0.57
**MMS (45 μM)**	63.33 ± 9.23*	38.00 ± 9.53*	56.00 ± 4.35*	5.33 ± 6.11	1.66 ± 1.52*	71.67 ± 17.01*

*Note:* DNA damage was expressed as “arbitrary units.” Control = DMSO 1%. One‐way analysis of variance and Dunnett's test.

^a^ Total number of damaged cells (class 1 + 2 + 3). Mean ± standard deviation of three replicates.

*Significantly different from negative control: *p < 0.01.

The data for the cytokinesis‐block micronucleus test and CBPI are presented in Tables 3 and 4. Statistical analysis of the data obtained showed that the two lowest concentrations of acylhydrazone tested were not clastogenic or aneugenic in either cell type. However, the highest tested concentration of 200 μg/mL produced a significant increase in binucleated lymphocytes with micronuclei, nucleoplasmic bridges, and nuclear buds, compared to the negative control. In HepG2/C3A cells, at this same concentration, an increase in micronucleated cells was also observed, although this increase was not statistically significant. Additionally, a reduction in the lymphocyte proliferation index was observed by the CBPI, at all three concentrations tested in lymphocytes, and at 100 μg/mL in HepG2/C3A cells. The positive control used on both cell types (MMS and benzo(a)pyrene) produced statistically significant increases in the number of binucleated cells with micronuclei, nucleoplasmic bridges, and nuclear buds.

**TABLE 3:** Micronucleus (MN) frequency, nucleoplasmic bridge (NPB), nuclear buds (NB), and cytokinesis‐block proliferation index (CBPI) in human peripheral blood leukocytes exposed to *N*′‐(3,5‐difluorobenzylidene)pyridine‐4‐carbohydrazide (in duplicate for each sample).

Test substance		Binucleated cells					
	Exposure (28 h)	With MN				CBPI	
	Concentrations	4000 cells		NPB	NB	500 cells	
	μg/mL	No.	%	No.	No.	Mean ± SD	
Negative control	0	12	0.30	10	20	2.06 ± 0.06	
MMS (positive control)	150^a^	69*	1.72*	59*	174*	1.83 ± 0.10*	
Test hydrazide	50	6	0.15	10	24	1.84 ± 0.05*	
	100	7	0.17	16	20	1.85 ± 0.04*	
	200	20*	0.50	43*	41*	1.83 ± 0.09*	

*Note:* Negative control: RPMI 1640 medium. One‐way analysis of variance (ANOVA) and Dunnett's test.

Abbreviation: SD = standard deviation.

^a^ Concentration = μM.

*Statistically different from the negative control: *p < 0.05.

**TABLE 4:** Micronucleus (MN) frequency, nucleoplasmic bridge (NPB), nuclear buds (NB), and cytokinesis‐block proliferation index (CBPI), in HepG2/C3A human cell line exposed to *N*′‐(3,5‐difluorobenzylidene)pyridine‐4‐carbohydrazide (in triplicate for each sample).

Test substance		Binucleated cells					
	Exposure (24 h)	with MN				CBPI	
	Concentrations	3000 cells		NPB	NB	500 cells	
	μg/mL	No.	%	No.	No.	Mean ± SD	
Negative control	0	3	0.10	6	11	1.59 ± 0.05	
Benzo(a)pyrene	2^a^	23*	0.76*	62*	39*	1.72 ± 0.01*	
Test hydrazide	50	0	0.00	9	7	1.57 ± 0.04	
	100	1	0.03	5	14	1.48 ± 0.01*	
	200	6	0.20	7	10	1.55 ± 0.01	

*Note:* Negative control: DMEM medium. One‐way analysis of variance (ANOVA), and Dunnett's test.

Abbreviation: SD = standard deviation.

^a^ Concentration = μM.

*Statistically different from the negative control: *p < 0.05.

## 4 Discussion

The N‐acylhydrazone moiety is considered a privileged structure and is present in many compounds exhibiting diverse pharmacological activities. Based on this structural feature, the *N*′‐(3,5‐difluorobenzylidene)pyridine‐4‐carbohydrazide was synthesized, and in this study, the cytotoxic and genotoxic potential of this new compound in human cells with and without hepatic metabolic capacity was initiated.

The first parameter that was investigated was cellular toxicity using the resazurin assay. Resazurin is a cell‐permeable redox indicator that can be used to monitor the number of viable cells. Viable cells with active metabolism can reduce resazurin (blue) to resorufin, a pink and fluorescent product (Riss et al. 2013). In the present study, concentrations ranging from 1 to 600 μg/mL were studied, after 24 and 48 h of exposure. In both cell types, a reduction in cell viability was observed only at 600 μg/mL, after 48 h of exposure. Nevertheless, cell viability remained above 85%.

The concentrations at which the biological effects of acylhydrazones occur vary according to the specific acylhydrazone structure, the biological target (bacteria, fungi, tumor cells, enzymes, etc.), and the assay type (in vitro vs. in vivo), but in general, the effects are observed within the range of 1 and 100 μg/mL (Socea et al. 2022). Frias et al. (2024) observed that the *N*′‐(3,5‐difluorobenzylidene)pyridine‐4‐carbohydrazide showed an IC_50_ for AChE inhibition of 2.73 μM (0.71 μg/mL). Therefore, the data obtained in the present study indicate that this acylhydrazone did not exhibit cytotoxicity because much higher concentrations were tested without a significant reduction in the viability of both cell types studied.

However, cytotoxic effects have been reported for some N‐acylhydrazones. The cytotoxic action was demonstrated for *N*′‐(1‐(4,7‐dihydroxy‐2‐oxo‐2H‐chromen‐3‐yl)ethylidene)benzohydrazide and *N*′‐(1‐(4‐hydroxy‐2‐oxo‐2H‐chromen‐3‐yl)ethylidene)benzohydrazide, where tubulin polymerization inhibition was shown to be the cause of the reduction in cell proliferation in vitro assays (Govindaiah et al. 2019). Aneja et al. (2019) observed that (*E*)‐1‐(4‐methoxybenzyl)‐*N*′‐(7‐methyl‐2‐oxoindolin‐3‐ylidene)‐1H‐1,2,3‐triazole‐4‐carbohydrazide exhibited inhibitory activity against microtubule affinity‐regulating kinase 4 (MARK4), a kinase involved in cell replication, producing an antiproliferative effect, increasing reactive oxygen species (ROS), and inducing apoptosis in cancer cell lines. Furthermore, the 5‐bromo‐1‐methyl‐*N*′‐[(*E*)‐(1‐methyl‐1H‐indol‐3‐yl)methylidene]‐1H‐indol‐3‐carbohydrazide exhibited antitumor activity in breast, cervical, and colon cancer cell lines by inducing apoptosis and stimulating the generation of ROS and nitric oxide (Sreenivasulu et al. 2019).

An additional aspect examined in this research was the capacity of *N*′‐(3,5‐difluorobenzylidene)pyridine‐4‐carbohydrazide to induce genotoxic responses in human cells. The comet assay and the micronucleus test were employed to achieve this goal. The comet assay, also known as single‐cell gel electrophoresis, is widely used due to its broad capacity to detect DNA damage (Singh et al. 1988; Araldi et al. 2015). The micronucleus test, which detects two mechanisms of chromosomal alterations: chromosomal breaks (clastogenesis) and disruption of the mitotic apparatus resulting in changes in chromosome number (aneugenesis), was also applied (Bonassi et al. 2007; Maistro 2014).

Our data obtained from the comet assay showed that none of the three concentrations of the tested acylhydrazone caused significant DNA damage in either cell type following 4 h of exposure. In contrast, the micronucleus test revealed significant chromosomal alterations in human lymphocytes at the highest concentration of 200 μg/mL, as well as cytotoxic effects, evidenced by a reduction in the CBPI in both cell types.

When considering the differences between the two cellular models employed, it is important to emphasize that HepG2/C3A cells possess hepatic metabolic capacity (Donato et al. 2015), whereas human lymphocytes (PBMC) lack this competence. This distinction is critical for interpreting the results. The preservation of cell viability in both models suggests that the acylhydrazone does not immediately compromise cellular survival. However, the increase in micronuclei and other nuclear alterations observed only in leukocytes at the highest tested concentration may indicate that the observed toxicity arises from direct interactions with genetic material or from metabolism‐independent cellular stress. This hypothesis warrants further investigation through complementary studies to clarify the underlying mechanisms.

Few specific genotoxicity studies have been conducted on acylhydrazones. Most of the literature describes general biological activities (antitumor, antimicrobial, anti‐inflammatory, etc.) rather than formal genotoxicity tests (Socea et al. 2022). Carcelli et al. (1995) reported a genotoxic evaluation of 2,6‐diacetylpyridine bis (acylhydrazones) and their complexes with some first‐transition‐series metal ions, using the in vitro *Bacillus subtilis* rec‐assay and the Ames test. None of the compounds tested were active in the rec‐assay, a test capable of detecting DNA‐damaging properties. However, in the Ames test, the ligands H2dapb, H2dapab, and H2dappc were active. Among the different ligands, H2dappc showed the highest mutagenic activity. The introduction of an amino group in H2dapb yielded H2dapab with higher mutagenic potency. On the other hand, according to the authors, the presence of a hydroxy group in the ortho position of the benzene ring (H2daps) suppressed the mutagenicity of 2,6‐diacetylpyridine benzoylhydrazone (Carcelli et al. 1995).

In another study, Bacchi et al. (1998) synthesized and evaluated the genotoxic effects of mono‐ and bimetallic organotin complexes with pyrrole‐2,5‐dicarboxaldehyde bis(2‐hydroxybenzoylhydrazone) and pyrrole‐2,5‐dicarboxaldehyde bis(2‐picolinoylhydrazone). None of the compounds induced DNA damage in the *B. subtilis* rec‐assay or exhibited mutagenic activity in the *Salmonella* microsome test.

The role of N‐acylhydrazones as ligands in medicinal inorganic chemistry has augmented the therapeutic potential and enhanced the biological activity of various metal and other complexes (Benitez et al. 2013; Thota et al. 2015; Mali et al. 2021; Socea et al. 2022). Similar to new compounds derived from the N‐acylhydrazone scaffold, which exhibit diverse biological activities, the few cytogenotoxic studies available in the literature also indicate varying cytotoxic and genotoxic potentials, depending on the ligands introduced and the methods employed. For compounds exhibiting antiproliferative effects, the mechanisms involved appear to include the formation of reactive oxygen species and the induction of apoptosis (Aneja et al. 2019; Govindaiah et al. 2019; Sreenivasulu et al. 2019). In the present study, a small reduction in cell proliferation, detected by CBPI analysis, was also observed, but the mechanisms underlying this effect require further investigation in future studies.

In summary, under the experimental conditions applied in this study, the findings suggest that the acylhydrazone *N*′‐(3,5‐difluorobenzylidene)pyridine‐4‐carbohydrazide did not elicit notable cytogenotoxic effects in human leukocytes or HepG2/C3A cells at lower concentrations, levels at which beneficial biological activities have previously been described. On the other hand, caution is warranted at high concentrations since chromosomal damage and a reduction in cell proliferation were observed. These findings support the continuation of studies aimed at developing this compound as a potential therapeutic agent, as well as assessing its safety in humans.

## Author Contributions

**Larissa Ribeiro Canuto Santos:** data collection and methodology. **Jéssica Natalia Barbosa de Almeida:** data collection and methodology. **Camila Cavalleiro Frias:** acylhydrazone synthesis. **Wanda Pereira Almeida:** responsible for acylhydrazone synthesis, manuscript review, and provision of resources. **Edson Luis Maistro:** conceptualization, project management, supervision, funding acquisition, and manuscript writing/review and editing.

## Funding

This work was supported by the Coordenação de Aperfeiçoamento de Pessoal de Nível Superior (Finance Code 001), Conselho Nacional de Desenvolvimento Científico e Tecnológico (303604/2021‐2), and Universidade Estadual Paulista.

## Conflicts of Interest

The authors declare no conflicts of interest.

## Data Availability

Data will be made available on request.

## References

[jat70171-bib-0001] AlNasser, M. N. , G. M. Alboraiy , E. M. Alsowig , and F. M. Alqattan . 2025. “Cholinesterase Inhibitors From Plants and Their Potential in Alzheimer's Treatment: Systematic Review.” Brain Sciences 15: 215. 10.3390/brainsci15020215. PMC11852592 40002547

[jat70171-bib-0002] Aneja, B. , N. S. Khan , P. Khan , et al. 2019. “Design and Development of Isatin‐Triazole Hydrazones as Potential Inhibitors of Microtubule Affinity‐Regulating Kinase 4 for the Therapeutic Management of Cell Proliferation and Metastasis.” European Journal of Medicinal Chemistry 163: 840–852. 10.1016/j.ejmech.2018.12.026. 30579124

[jat70171-bib-0003] Antoniou, E. E. , R. Martijn , A. K. Neslihan , M. N. Colin , and P. Z. Maurice . 2023. “Micronuclei in Human Peripheral Blood and Bone Marrow as Genotoxicity Markers: A Systematic Review and Meta‐Analysis.” Mutation Research, Genetic Toxicology and Environmental Mutagenesis 891: 503–689. 10.1016/j.mrgentox.2023.503689. 37770146

[jat70171-bib-0004] Araldi, R. P. , T. C. de Melo , T. B. Mendes , et al. 2015. “Using the Comet and Micronucleus Assays for Genotoxicity Studies: A Review.” Biomedicine & Pharmacotherapy 72: 74–82. 10.1016/j.biopha.2015.04.004. 26054678

[jat70171-bib-0005] Bacchi, A. , A. Bonardi , M. Carcelli , et al. 1998. “Organotin Complexes With Pyrrole‐2,5‐Dicarboxaldehyde Bis (Acylhydrazones): Synthesis, Structure, Antimicrobial Activity and Genotoxicity.” Journal of Inorganic Biochemistry 69: 101–112. 10.1016/S0162-0134(97)10027-7. 9606941

[jat70171-bib-0006] Benitez, J. , A. Cavalcanti de Queiroz , I. Correia , et al. 2013. “New oxidovanadium (IV) N‐Acylhydrazone Complexes: Promising Antileishmanial and Antitrypanosomal Agents.” European Journal of Medicinal Chemistry 62: 20–27. 10.1016/j.ejmech.2012.12.036. 23353731

[jat70171-bib-0007] Bonassi, S. , A. Znaor , M. Ceppi , C. Lando , W. Chang , and N. Holland . 2007. “An Increased Micronucleus Frequency in Peripheral Blood Lymphocytes Predicts the Risk of Cancer in Humans.” Carcinogenesis 28: 625–631. 10.1093/carcin/bgl177. 16973674

[jat70171-bib-0008] Brum, J. O. C. , T. C. C. França , S. R. LaPlante , and J. D. F. Villar . 2020. “Synthesis and Biological Activity of Hydrazones and Derivatives: A Review.” Mini‐Reviews in Medicinal Chemistry 20: 342–368. 10.2174/1389557519666190912150051. 31612828

[jat70171-bib-0009] Carcelli, M. , P. Mazza , C. Pelizzi , G. Pelizzi , and F. Zani . 1995. “Antimicrobial and Genotoxic Activity of 2,6‐Diacetylpyridine Bis (Acylhydrazones) and Their Complexes With Some First Transition Series Metal Ions: X‐Ray Crystal Structure of a Dinuclear Copper (II) Complex.” Journal of Inorganic Biochemistry 57: 43–62. 10.1016/0162-0134(94)00004-T. 7876834

[jat70171-bib-0010] Donato, M. T. , L. Tolosa , and M. J. Gómez‐Lechón . 2015. “Culture and Functional Characterization of Human Hepatoma HepG2/C3A Cells.” In Human Cell Culture Protocols (Methods in Molecular Biology, vol. 1250). Springer. 10.1007/978-1-4939-2074-7_5. 26272135

[jat70171-bib-0011] Ellinger‐Ziegelbauer, H. , J. Aubrecht , J. C. Kleinjans , and H. J. Ahr . 2009. “Application of Toxicogenomics to Study Mechanisms of Genotoxicity and Carcinogenicity.” Toxicology Letters 186: 36–44. 10.1016/j.toxlet.2008.08.017. 18822359

[jat70171-bib-0012] Fenech, M. 2000. “The In Vitro Micronucleus Technique.” Mutation Research 455: 81–95. 10.1016/s0027-5107(00)00065-8 11113469

[jat70171-bib-0013] Frias, C. C. , G. Antoniolli , W. P. Barros , and W. P. Almeida . 2024. “Acylhydrazones Derived From Isonicotinic Acid: Synthesis, Characterization, and Evaluation Against Alzheimer's Disease Biomarkers.” Journal of Molecular Structure 1313: 138631. 10.1016/j.molstruc.2024.138631.

[jat70171-bib-0014] Govindaiah, P. , N. Dumala , I. Mattan , P. Grover , and M. Jaya Prakash . 2019. “Design, Synthesis, Biological and In Silico Evaluation of Coumarin‐Hydrazone Derivatives as Tubulin‐Targeted Antiproliferative Agents.” Bioorganic Chemistry 91: 103143. 10.1016/j.bioorg.2019.103143. 31374528

[jat70171-bib-0015] Hartmann, A. , and G. Speit . 1997. “The Contribution of Cytotoxicity to DNA Effects in the Single Cell Gel Test (Comet Assay).” Toxicology Letters 90: 183–188. 10.1016/S0378-4274(96)03847-7. 9067486

[jat70171-bib-0016] Hewitt, N. J. , and P. Hewitt . 2004. “Phase I and II Enzyme Characterization of Two Sources of HepG2 Cell Lines.” Xenobiotica 34: 243–256. 10.1080/00498250310001657568. 15204697

[jat70171-bib-0017] Knasmüller, S. , V. Mersch‐Sundermann , S. Kevekordes , et al. 2004. “Use of Human‐Derived Liver Cell Lines for the Detection of Environmental and Dietary Genotoxicants: Current State of Knowledge.” Toxicology 198: 315–328. 10.1016/j.tox.2004.02.008. 15138058

[jat70171-bib-0018] Lebailly, P. , M. Gladys , H. Fabrice , L. Yannick , S. Bernard , and B. R. Elisa . 2015. “DNA Damage in B and T Lymphocytes of Farmers During One Pesticide Spraying Season.” International Archives of Occupational and Environmental Health 88: 963–972. 10.1007/s00420-015-1024-3. PMC4564440 25647545

[jat70171-bib-0019] Maistro, E. L. 2014. “The In Vivo Rodent Micronucleus Test.” In Genotoxicity and DNA Repair: A Practical Approach, edited by L. M. Sierra and I. Gaivão , 103–113. Springer Science & Business Media.

[jat70171-bib-0020] Mali, S. N. , B. R. Thorat , D. R. Gupta , and A. Pandey . 2021. “Mini‐Review of the Importance of Hydrazides and Their Derivatives—Synthesis and Biological Activity.” Engineering Proceedings 11: 21. 10.3390/ecsoc-24-11764.

[jat70171-bib-0021] OECD . 2016a. “Test No. 489: In Vivo Mammalian Alkaline Comet Assay.” In OECD Guidelines for the Testing of Chemicals, Section 4. OECD Publishing. 10.1787/9789264264885-en.

[jat70171-bib-0022] OECD . 2016b. “Test No. 487: In Vitro Mammalian Cell Micronucleus Test.” In OECD Guidelines for the Testing of Chemicals, Section 4. OECD Publishing. 10.1787/9789264264878-en.

[jat70171-bib-0023] Riss, T. L. , R. A. Moravec , and A. L. Niles . 2013. “Cell Viability Assays.” In Assay Guidance Manual [Internet], edited by I. G. S. Sittampalam , A. Grossman , and K. Brimacombe . Eli Lilly & Company and the National Center for Advancing Translational Sciences. https://www.ncbi.nlm.nih.gov/books/.

[jat70171-bib-0024] Saud, A. , V. Krishnaraju , A. Taha , et al. 2024. “Potential Acetylcholinesterase Inhibitors to Treat Alzheimer's Disease.” European Review for Medical and Pharmacological Sciences 28: 2522–2537. 10.26355/eurrev_202403_35759 38567612

[jat70171-bib-0025] Secerli, J. , Ş. Çelikten , O. Erdem , and M. Güdül Bacanli . 2024. “The Relationship Between Genotoxicity and Cancer.” In Pathophysiology of Cancer: An Interdisciplinary Approach (Interdisciplinary Cancer Research, 22), edited by N. Rezaei . Springer. 10.1007/16833_2024_335.

[jat70171-bib-0026] Singh, N. P. , M. T. McCoy , R. R. Tice , and E. L. Schneider . 1988. “A Simple Technique for Quantitation of Low Levels of DNA Damage in Individual Cells.” Experimental Cell Research 175: 184–191. 10.1016/0014-4827(88)90265-0. 3345800

[jat70171-bib-0027] Socea, L. I. , S. F. Barbuceanu , E. M. Pahontu , et al. 2022. “Acylhydrazones and Their Biological Activity: A Review.” Molecules 27, no. 24: 8719. 10.3390/molecules27248719. PMC9783609 36557851

[jat70171-bib-0028] Sreenivasulu, R. , K. T. Reddy , P. Sujitha , C. G. Kumar , and R. R. Raju . 2019. “Synthesis, Antiproliferative and Apoptosis Induction Potential Activities of Novel Bis (Indolyl)Hydrazide‐Hydrazone Derivatives.” Bioorganic & Medicinal Chemistry 27: 1043–1055. 10.1016/j.bmc.2019.02.002. 30773423

[jat70171-bib-0029] Sugiura, M. , and S. Kobayashi . 2005. “N‐Acylhydrazones as Versatile Electrophiles for the Synthesis of Nitrogen‐Containing Compounds.” Angewandte Chemie International Edition 44: 5176–5186. 10.1002/anie.200500691. 16059954

[jat70171-bib-0030] Thota, S. , D. A. Rodrigues , P. d. S. M. Pinheiro , L. M. Lima , C. A. M. Fraga , and E. J. Barreiro . 2018. “N‐Acylhydrazones as Drugs.” Bioorganic & Medicinal Chemistry Letters 28: 2797–2806. 10.1016/j.bmcl.2018.07.015. 30006065

[jat70171-bib-0031] Thota, S. , S. Vallala , R. Yerra , and E. J. Barreiro . 2015. “Design, Synthesis, Characterization, Cytotoxic and Structure–Activity Relationships of Novel Ru (II) Complexes.” Chinese Chemical Letters 26: 721–726. 10.1016/j.cclet.2015.03.011.

[jat70171-bib-0032] Tice, R. R. , E. Agurell , D. Anderson , et al. 2000. “Single Cell Gel/Comet Assay: Guidelines for In Vitro and In Vivo Genetic Toxicology Testing.” Environmental and Molecular Mutagenesis 35: 206–221. 10.1002/(SICI)1098-2280(2000)35:3<206::AID-EM8>3.0.CO;2-J. 10737956

